# Application of holmium laser in endoscopic retrograde cholangiopancreatographygall gallbladder-preserving cholecystolithotomy

**DOI:** 10.1055/a-2499-7536

**Published:** 2025-02-06

**Authors:** Qian Zou, Jingfeng Du, Yaosheng Lin, Long Xu

**Affiliations:** 1 558113 Department of Gastroenterology and Hepatology, Shenzhen University General Hospital, Shenzhen, China; 2 47890 Marshall Laboratory of Biomedical Engineering, Shenzhen University, Shenzhen, China

We report a case of a 59-year-old woman diagnosed with cholecystolithiasis ( Fig. 1 ). She frequently experienced discomfort in the right upper abdomen after consuming greasy foods but was reluctant to undergo cholecystectomy. Consequently, she sought our assistance and requested a gallbladder-preserving cholecystolithotomy. Following thorough communication, the decision was made to proceed with endoscopic retrograde cholangiopancreatography (ERCP)-based gallbladder-preserving cholecystolithotomy.

**Fig. 1:**
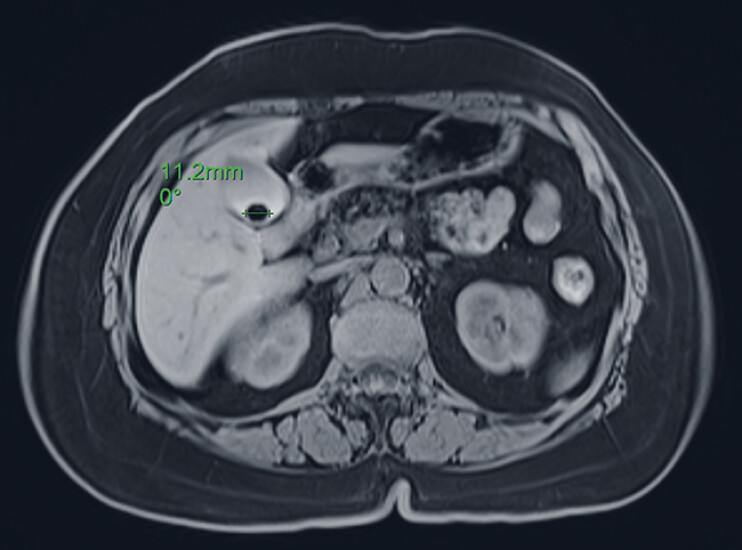
Magnetic resonance imaging of the gallstone.

After placement of the pancreatic stent, biliary duct stent, and two fully covered metal stents (FCMSs) (one of which extended into the gallbladder cavity), a gallstone approximately 11 mm in diameter was observed when the choledochoscope entered into the gallbladder cavity. However, the gallstone was too large and hard to remove through the FCMS ( Fig. 2 ), and it could not be fragmented by electrohydraulic lithotripsy. Consequently, holmium laser lithotripsy was used ( Fig. 3 ). The gallstone was subsequently crushed and extracted by a reticular basket. Two days later, a choledochoscopy reticular basket was used to remove the remaining gallstone fragments from the gallbladder cavity. After that, the gallbladder was thoroughly rinsed. Finally, all the stents were removed, and a double-pigtail plastic stent was placed between the gallbladder and the duodenum for drainage ( Video 1 ). There was no significant discomfort or complication after treatment, and the double-pigtail plastic stent was removed by gastroscope in the outpatient clinic 1 month later ( Fig. 4 ). No recurrence was found during the 13-month follow-up.

**Fig. 2:**
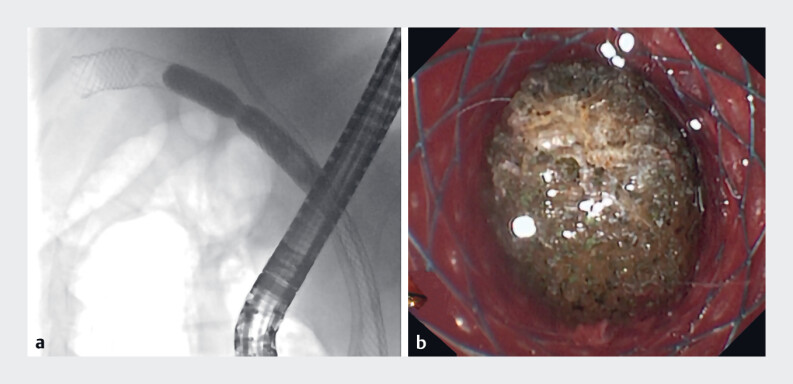
**a** The gallstone shown on X-ray. **b** The gallstone observed through choledochoscopy.

**Fig. 3:**
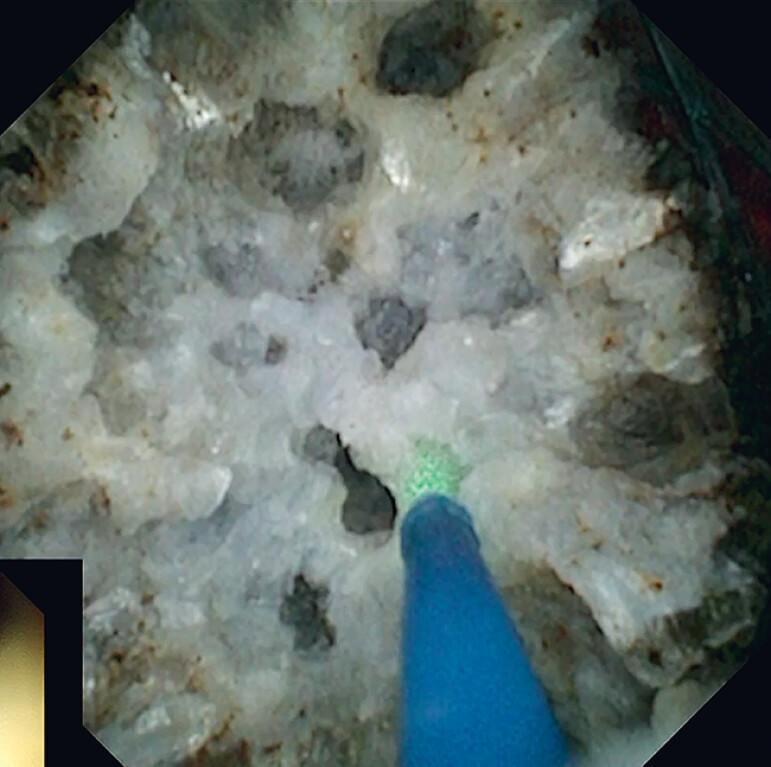
Holmium laser lithotripsy under direct vision of the choledochoscope.

**Fig. 4:**
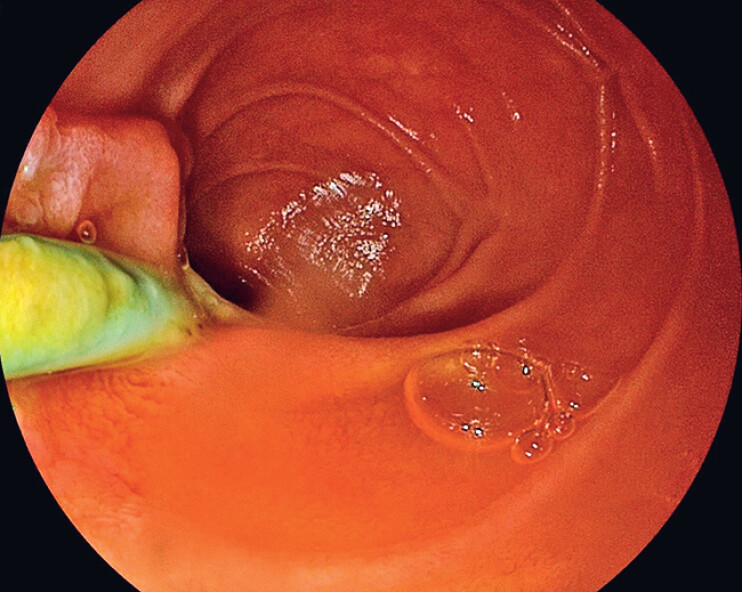
The double-pigtail plastic stent was removed by gastroscope 1 month later.

Application of a holmium laser in endoscopic retrograde cholangiopancreatography-based gallbladder-preserving cholecystolithotomy.Video 1

The gallbladder plays a crucial role in the digestive system, and complications such as abdominal pain and diarrhea may arise following cholecystectomy. ERCP-based gallbladder-preserving cholecystolithotomy can preserve the integrity and even functionality of the gallbladder after gallstone removal, making it a viable treatment option 1 . For large stones in the gallbladder, holmium laser lithotripsy under direct visualization with the choledochoscope is also an effective alternative.

Endoscopy_UCTN_Code_TTT_1AR_2AH

## Notes

Endoscopy E-Videos https://eref.thieme.de/e-videos
*E-Videos* is an open access online section of the journal *Endoscopy* , reporting on interesting cases and new techniques in gastroenterological endoscopy. All papers include a high-quality video and are published with a Creative Commons CC-BY license. Endoscopy E-Videos qualify for HINARI discounts and waivers and eligibility is automatically checked during the submission process. We grant 100% waivers to articles whose corresponding authors are based in Group A countries and 50% waivers to those who are based in Group B countries as classified by Research4Life (see: https://www.research4life.org/access/eligibility/ ). This section has its own submission website at https://mc.manuscriptcentral.com/e-videos .
